# Saliva proteomic patterns in patients with molar incisor hypomineralization

**DOI:** 10.1038/s41598-020-64614-z

**Published:** 2020-05-05

**Authors:** K. Bekes, G. Mitulović, N. Meißner, U. Resch, R. Gruber

**Affiliations:** 1 Department of Paediatric Dentistry, School of Dentistry, Medical University of Vienna, Vienna, Austria; 2 Proteomics Core Facility, Clinical Institute of Laboratory Medicine, Medical University of Vienna, Vienna, Austria; 3 Private practice, Salzburg, Austria; 4 Department of Vascular Biology and Thrombosis Research, Medical University of Vienna, Vienna, Austria; 5 Department of Oral Biology, School of Dentistry, Medical University of Vienna, Vienna, Austria; 6 Department of Periodontology, School of Dental Medicine, University of Bern, Bern, Switzerland; 7 Austrian Cluster for Tissue Regeneration, Vienna, Austria

**Keywords:** Dental diseases, Paediatric research, Proteomics

## Abstract

Molar incisor hypomineralization (MIH) is an endemic pediatric disease with an unclear pathogenesis. Considering that saliva controls enamel remineralization and that MIH is associated with higher saliva flow rate, we hypothesized that the protein composition of saliva is linked to disease. To test this, we enrolled 5 children aged 6–14 years with MIH showing at least one hypersensitive molar and 5 caries-free children without hypomineralization. Saliva samples were subjected to proteomic analysis followed by protein classification in to biological pathways. Among 618 salivary proteins identified with high confidence, 88 proteins were identified exclusively in MIH patients and 16 proteins in healthy controls only. Biological pathway analysis classified these 88 patient-only proteins to neutrophil-mediated adaptive immunity, the activation of the classical pathway of complement activation, extracellular matrix degradation, heme scavenging as well as glutathione -and drug metabolism. The 16 controls-only proteins were associated with adaptive immunity related to platelet degranulation and the lysosome. This report suggests that the proteaneous composition of saliva is affected in MIH patients, reflecting a catabolic environment which is linked to inflammation.

## Introduction

The term molar incisor hypomineralization (MIH) was coined in 2001 and defines a qualitative enamel defect affecting one or more first permanent molars with or without the involvement of permanent incisors^1^. The global prevalence of MIH exceeds one-tenth of children, ranging from 0.5% to 40.2% and differing between countries^2^. Demarcated hypomineralized enamel lesions are caused by the process of amelogenesis being altered or interrupted^3^. Compared to normal teeth, affected enamel is characterized by a reduction in mineral quantity and quality, increased porosity and reduced hardness^4^. The clinical management of MIH is challenging because of rapid wear, progressing enamel loss, increased susceptibility to caries, loss of fillings, and severe hypersensitivity^5^. Although MIH is a pandemic pediatric disease, the etiology remains unknown^6^. Several hypotheses are proposed, including childhood illness, genetic influences^7,8^, and a putative individual threshold of susceptibility^3^. Thus, there is an increasing demand to better understand the cause and consequence(s) of MIH at the cellular and molecular level.

Chronic subclinical pulpal inflammation is a consequence of increased enamel porosity and reduced hardness in MIH^9^. Pulpitis is characterized by enhanced neutrophil emigration into the pulp tissue^10,11^ and biomarkers can be identified in gingival crevicular fluid^12^. Moreover, in periodontitis patients, neutrophils constantly migrate through the oral epithelia into the saliva^13^, with increasing numbers exhibiting apoptosis and augmented levels of degranulation markers^14^. Further, saliva of MIH patients reportedly displays altered physicochemical properties such as altered flow rates, viscosity, pH and acid buffering capacity^15^. Thus, it is conceivable that the protein-composition of MIH saliva may exhibit characteristic changes that cause or contribute to the clinical symptoms of this disease.

Salivary proteome analysis has progressively evolved in various biomedical disciplines such as genetics, molecular biology, medicine, and dentistry^16^ in the last decade^17,18^. The salivary proteome has been exploited to detect oral diseases such as periodontitis^19^, oral squamous cell carcinoma^20,21^, burning mouth syndrome^22^ and Sjögren’s syndrome^23^. Moreover, the saliva proteome was analyzed in systemic diseases, e.g. diabetes mellitus^24^, cystic fibrosis^25^, Parkinson disease^26^, pulmonary tuberculosis^27^, multiple sclerosis^28^, Zika virus^29^ as well as in psychiatric^30^ and genetic diseases^31^, proving the great potential of proteomics in both biomarker identification and providing insight into the molecular mechanisms underlying disease pathology. Saliva is increasingly used for liquid biopsy applications^32–34^. Saliva is also used for screening of biomarkers e.g. in colorectal cancer^35–37^ or systolic heart failure^38^. As changes in saliva composition are associated with oral diseases, it is reasonable to assume that compared to healthy children, the saliva of MIH children experiencing subclinical inflammation might be altered. Here, we employed high-resolution shotgun proteomics to identify protein signatures unique to MIH patients.

## Methods

### Study design and population

This cohort study was undertaken in caries-free children with and without MIH. Patients were recruited from the Department of Paediatric Dentistry, School of Dentistry, Medical University of Vienna, Austria. The study population included 5 children with MIH showing hypersensitivity, compared with a control group of healthy peers all under 14 years of age. For the MIH group, criteria proposed by the European Academy of Paediatric Dentistry (EAPD)^39^ was used for MIH diagnosis including; the presence of demarcated opacities, post-eruptive enamel breakdown, atypical restorations and extraction due to MIH in at least one first permanent molar. Demarcated opacities with a diameter of <1 mm were not considered in the analysis. Furthermore, MIH teeth were graded using the MIH-TNI (MIH Treatment Need Index)^40^. Inclusion criteria were children and adolescents aged 6–14 years, at least one hypersensitive molar with MIH which had a qualifying response to air blast stimuli applied for one second as defined by a score of 2 or 3 on the Schiff Cold Air Sensitivity Scale. Exclusion criteria were systemic diseases, long-term medication, hypomineralized molar due to other medical conditions, hypersensitive study teeth with contributing etiologies other than recognized clinically as being associated with MIH, caries or restorations in study teeth. For the control group, caries-fee children of the same age group with no MIH were included applying the same inclusion and exclusion criteria.

### Ethical aspects and saliva collection

Approval for this clinical investigation was obtained from the ethics committee of the local University Review Board (Medical University of Vienna; Approval: 1463/2016). Written statements of consent were read and signed by children and their guardians prior to their participation and all experiments were performed in accordance with relevant guidelines and regulations of the Ethics Committee of the Medical University of Vienna. Study subjects were instructed to refrain from all oral hygiene procedures, chewing gum and painkillers for 8 h and from eating, drinking and brushing for 1 h prior to examinations^41,42^. Saliva collection was performed using Salivette (Sarstedt, Germany). Each patient was instructed to chew the swab for 120 seconds to stimulate salivation. Afterwards, the swab with the absorbed saliva was returned to the Salivette^43^. Samples were centrifuged for 10 min, 10000 rpm, 4 °C, and stored at −20 °C until further use.

### Mass spectrometry

Saliva proteins were precipitated using methanol/dichloromethane and digested with trypsin as described earlier^44^ (For detail see Suppl. Methods 1). Precipitated proteins were dissolved in 0.1% Rapigest (Waters, Vienna, Austria), dissolved in 50 mM triethylammonium bicarbonate, and protein concentration was determined using the Bradford assay. Proteins were digested overnight at 37 °C using a trypsin:protein ratio of 1:50, digestion was stopped by acidification with trifluoroacetic acid (TFA). Following injection onto the trapping column (Acclaim C18 trap column, 300μm inner diameter × 5 mm), peptides were separated by nano-reverse-phase (Acclaim C18, 75μm inner diameter × 500 mm) using an UltiMate nano RSLC HPLC (Thermo Fisher, Germering, Germany) separation system, consisting of the autosampler, column switching unit, nano and loading pump and UV detector. Both, trap- and separation columns were operated at 60 °C and UV peptide detection at 214 nm served as quality control for HPLC separation. Samples were loaded onto the trap column using 0.1% TFA at 30 μl/min and precooled to 3 °C^45^, nano separation was performed in gradient mode at 300 nl/min. A user defined injection program was used for sample injection and additional injector and trap column wash. Every sample injection was followed by two blank runs with injections of 2,2,2-trifluoroethanol for removal of possible sample remains in the injector or on the trap column and prevention of carryover in the separation system. Mass spectrometry (MS) analysis was performed using the Q-Exactive plus mass spectrometer (Thermo Fisher Scientific) and the “top 20” method for MS/MS experiment; that is, the 20 most intensive ions from the MS scan were selected for tandem MS (MS/MS), single-charged ions were excluded from fragmentation, and detected ions were excluded for further fragmentation for 2 min after initial MS/MS fragmentation had been performed. Mass resolution of 70000 was selected for MS at AGC set to 3E6 ions, MS/MS resolution was set to 35000 and AGC set to 1E5 ions. Fragmentation was performed using the HCD approach at normalized collision energy of 30 eV. Data analysis (database search and label-free quantitation) was performed using MaxQuant (version 1.6.0.1) with following parameters: MS/MS data were searched against the Human Fasta Database (Uniprot, version September 2018), MS/MS tolerance was set to 20ppm, deamidation on N and Q, N-term acetylation and oxidation on M were selected as variable modifications. The mass spectrometry proteomics data have been deposited to the ProteomeXchange Consortium via the PRIDE partner repository with the dataset identifier PXD016126^46^.

### Statistical analysis and data visualization

Bioinformatic analyses of protein identifications were done in Perseus (version 1.5.5.3) and overlapping proteins were visualized in Venn-Diagrams in combination with the CNB-CSIC online tool *Venny*^47^. Gene-Ontology as well as biological pathway enrichment analysis were done using the “Enricher” interactive data-analysis tool^48,49^. Throughout this report we express enriched terms by enumerating the actual number of proteins in our dataset in relation to the total number of proteins consolidated for the respective pathway. Normalizations were done using the web-based tool NormalyzerDE^50^. Spearman-rank correlation analysis and visualization of log2-transformed label-free quantification (LFQ) values, unsupervised hierarchical clustering and heat-maps of median, quantile and rank-normalized protein abundance values were done in Perseus using default settings (euclidian-distance on average linkage, pre-processing with k-means with a maximum of 300 clusters and 10 iterations). For group-wise comparisons we employed FDR-based 1-Way-ANOVA with 250 permutations and multiple-testing correction of p-values were done in InstantClue (version 0.5.2) using non-log transformed p-values for 2-stage-set-up Benjamini-Krieger-Yekutieli, alpha-error of 0.05 as well as Benjamini-Hochberg correction methods^51^. Volcano-plots were prepared in Perseus and proteins with a corrected p-value <0.05 were annotated with the corresponding gene symbol, while proteins with a non-adjusted p-value <0.05 were color-indicated only. For rank-normalization, only proteins with a corrected p-value smaller than 0.015 were annotated. For proteins exclusively present in patients or controls, a (−)log10-p-value of zero was assigned after all statistical analysis to enable visualization in Volcano plots. Visualization of protein–protein associations was performed with STRING v11.

## Results

### Patient characteristics

In the MIH group, 4 female and 1 male patient with a sum of 23 affected teeth were included (mean age 8.70 ± 2.36 years). The caries-free control group included 3 male and 2 females (mean age 10.73 ± 0.54 years). MIH patients showed three up to four affected first permanent molars with at least one tooth exhibiting hypersensitivity. Detailed study-cohort characteristics are shown in Supplementary Table 1. In total, 87.5% of the teeth were hypersensitive with a mean Schiff Score of 2.3 ± 0.48 and a VAS of 7.03 ± 2.14.

### Proteomic analysis

**Figure 1:**
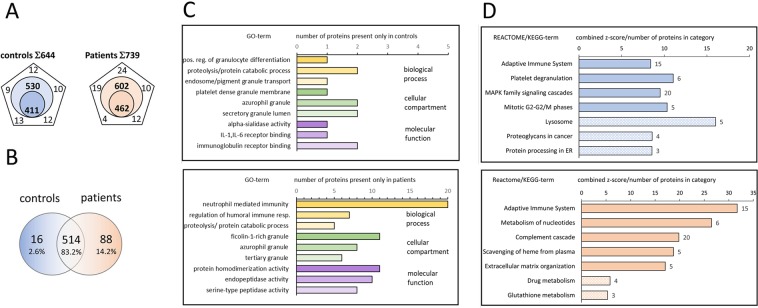
Qualitative comparison of saliva-proteins identified in controls and MIH-patients. (**A**) Exclusive and common proteins identified by at least two unique peptides in controls and MIH-patients, respectively. Total number of distinct proteins quoted at the top of the pentagon, numbers inscribed in the corners are unique proteins in individual samples. The inscribed smaller circle quotes proteins commonly identified in all samples, numbers in the bigger circle quotes protein-numbers identified in at least 3 out of 5 controls and MIH-patients, respectively. (**B**) Venn-diagram depicting numbers and percentage of common and exclusive proteins identified in saliva of controls or MIH-patients. (**C**) Significantly enriched Gene-Ontology (GO) biological process, cellular compartment and molecular function-terms of exclusive (“only”) proteins in controls (upper panel) or MIH-patients (lower panel). Protein-counts for respective terms are shown on the x-axis. (**D**) Enriched biological pathway-terms (REACTOME, filled bars; KEGG, dashed bars) and respective protein-counts (numbers at the bar-edges) for proteins found only in controls (upper panel) or MIH-patients (lower panel).

**Figure 2:**
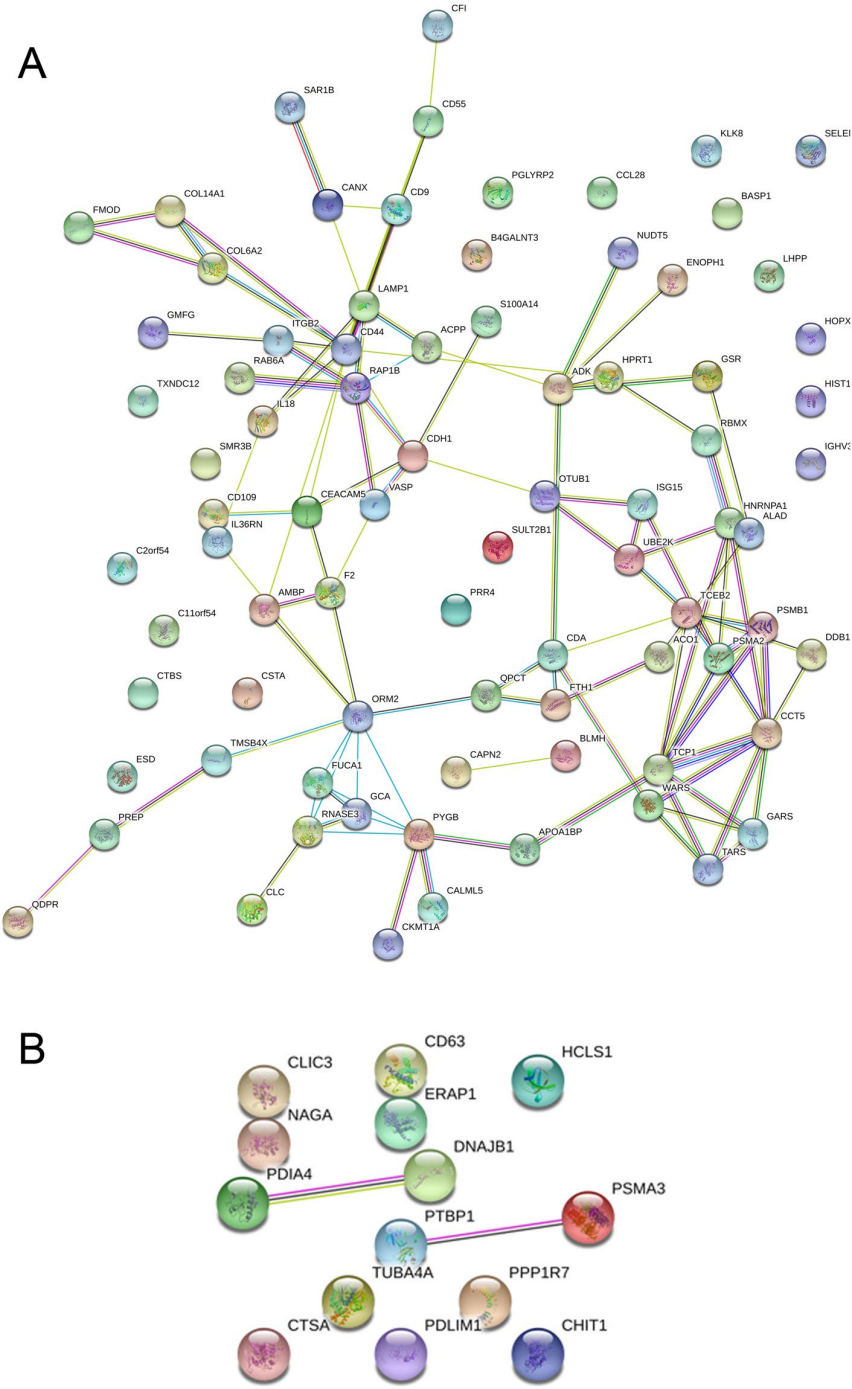
(**A**) STRING protein-protein interaction networks functional enrichment analysis of exclusive proteins identified by at least two unique peptides in MIH-patients. Number of nodes: 81; number of edges: 105; average node degree: 2.59; avg. local clustering coefficient: 0.36. (**B**) STRING protein-protein interaction networks functional enrichment analysis of exclusive proteins identified by at least two unique peptides in healthy control patients. Number of nodes: 14; number of edges: 2; average node degree: 0.286; avg. local clustering coefficient: 0.286.

In our high-resolution shotgun proteomic analysis, 462 (602) and 411 (530) proteins, identified and quantified with at least 2 peptides, were present in all 5 subjects or in at least 3 out of 5 subjects (numbers in parenthesis) within the patients and control group, respectively (Fig. 1A). Analysis of overlapping proteins among patients and controls is shown in Fig. 1B. The vast majority of proteins (514, 83.2%) were present in both patients and controls, however, 88 (14.2%) proteins were exclusively present in patient’s saliva and 16 (2.6%) in controls only (Fig. 1B, Table 1 and Table 2). Visualization of the protein–protein associations showed 81 nodes and 105 edges on the proteins exclusively detected in MIH saliva, whereas 14 nodes and only 2 edges were shown for saliva of healthy controls (Fig. 2).

**Table 1:** Exclusive proteins identified by at least two unique peptides in MIH-patients.

Gene Symbol	Protein description
ACO1	aconitase 1
ACPP	acid phosphatase, prostate
ADK	adenosine kinase
ALAD	aminolevulinate dehydratase
AMBP	alpha-1-microglobulin/bikunin precursor
APOA1BP	NAD(P)HX epimerase
BASP1	brain abundant membrane attached signal protein 1
BLMH	bleomycin hydrolase
C11orf54	chromosome 11 open reading frame 54
C2orf54	chromosome 2 open reading frame 54
CALML5	calmodulin like 5
CANX	calnexin
CAPN2	calpain 2
CCL28	C-C motif chemokine ligand 28(
CCT5	chaperonin containing TCP1 subunit 5
CD109	CD109 molecule
CD44	CD44 molecule (Indian blood group)
CD55	CD55 molecule (Cromer blood group)
CD9	CD9 molecule
CDA	cytidine deaminase
CDH1	cadherin 1
CEACAM5	carcinoembryonic antigen related cell adhesion molecule 5
CFI	complement factor I
CKMT1A	creatine kinase, mitochondrial 1B
CLC	Charcot-Leyden crystal galectin
COL14A1	collagen type XIV alpha 1 chain
COL6A2	collagen type VI alpha 2 chain
CSTA	cystatin A
CTBS	chitobiase
DDB1	damage specific DNA binding protein 1
ENOPH1	enolase-phosphatase 1
ESD	esterase D
F2	coagulation factor II, thrombin
FMOD	fibromodulin
FTH1	ferritin heavy chain 1
FUCA1	fucosidase, alpha-L- 1, tissue
GARS	glycyl-tRNA synthetase
GCA	grancalcin
GMFG	glia maturation factor gamma
GSR	glutathione-disulfide reductase
HIST1H2BJ	histone cluster 1 H2B family member j
HNRNPA1	heterogeneous nuclear ribonucleoprotein A1
HOPX	HOP homeobox
HPRT1	hypoxanthine phosphoribosyltransferase 1
IGHD	immunoglobulin heavy constant delta
IGHV1–69	immunoglobulin heavy variable 1–69
IGHV3–15	immunoglobulin heavy variable 3–15
IGKV1–5	immunoglobulin kappa variable 1–5
IGKV1D-39	immunoglobulin kappa variable 1D-39
IGKV2D-24	immunoglobulin kappa variable 2D-24 (non-functional)
IGKV4–1	immunoglobulin kappa variable 4–1
IGLV8–61	immunoglobulin lambda variable 8–61
IL18	interleukin 18
IL36RN	interleukin 36 receptor antagonist
ISG15	ISG15 ubiquitin-like modifier
ITGB2	integrin subunit beta 2
KLK8	kallikrein related peptidase 8
LAMP1	lysosomal associated membrane protein 1
LHPP	phospholysine phosphohistidine inorganic pyrophosphate phosphatase
NUDT5	nudix hydrolase 5
ORM2	orosomucoid 2
OTUB1	OTU deubiquitinase, ubiquitin aldehyde binding 1
PGLYRP2	peptidoglycan recognition protein 2
PREP	prolyl endopeptidase
PRR4	proline rich 4 (lacrimal)
PSMA2	proteasome subunit alpha 2
PSMB1	proteasome subunit beta 1
PYGB	phosphorylase, glycogen; brain
QDPR	quinoid dihydropteridine reductase
QPCT	glutaminyl-peptide cyclotransferase
RAB6A	RAB6A, member RAS oncogene family
RAP1B	RAP1B, member of RAS oncogene family
RBMX	RNA binding motif protein, X-linked
RNASE3	ribonuclease A family member 3
S100A14	S100 calcium binding protein A14
SAR1B	secretion associated Ras related GTPase 1B
SELENBP1	selenium binding protein 1
SMR3B	submaxillary gland androgen regulated protein 3B
SULT2B1	sulfotransferase family 2B member 1
TARS	threonyl-tRNA synthetase
TCEB2	transcription elongation factor B subunit 2
TCP1	t-complex 1
TMSB4X	thymosin beta 4, X-linked
TXNDC12	thioredoxin domain containing 12
UBE2K	ubiquitin conjugating enzyme E2 K
VASP	vasodilator-stimulated phosphoprotein
WARS	tryptophanyl-tRNA synthetase

**Table 2:** Exclusive proteins identified by at least two unique peptides in healthy patients.

Gene Symbol	Protein description
CD63	CD63 molecule
CHIT1	chitinase 1
CLIC3	chloride intracellular channel 3
CTSA	cathepsin A
DNAJB1	DnaJ heat shock protein family (Hsp40) member B1
ERAP1	endoplasmic reticulum aminopeptidase 1
HCLS1	hematopoietic cell-specific Lyn substrate 1
IGHV2–70D	Immunoglobulin heavy variable 2–70D
IGHV3OR15–7	immunoglobulin heavy variable 3/OR15–7 (pseudogene)
NAGA	alpha-N-acetylgalactosaminidase
PDIA4	protein disulfide isomerase family A member 4
PDLIM1	PDZ and LIM domain 1
PPP1R7	protein phosphatase 1 regulatory subunit 7
PSMA3	proteasome subunit alpha 3
PTBP1	polypyrimidine tract binding protein 1
TUBA4A	tubulin alpha 4a

### Label-free quantification (LFQ)

Applying an LFQ algorithm on our proteomic data, we found good individual correlations among controls (mean r = 0.866 ± 0.036) and patients (mean r = 0.829 ± 0.0518), although patient heterogeneity was higher (Supplementary Fig. 1A). We also noticed that LFQ-intensities of proteins within the patient´s group were generally higher, impeding comparative statistical analysis without prior normalization. To this end, we performed median and quantile-normalizations as well as rank-normalization, which achieves robustness to non-additive noise at the expense of losing parametric information on abundance values^50,52^. To ensure a highly reliable dataset, we omitted imputation of missing values which is applied in many proteomics studies.

### Hierarchical clustering of abundance values

Unsupervised hierarchical clustering of abundance values found in all samples clearly separated patients from controls (Supplementary Fig. 2A–C). Pertinent statistical evaluation of differentially expressed proteins obtained by FDR-based 1-way ANOVA following each normalization procedure is illustrated in Volcano-plots, showing expression differences (fold change) and depicting proteins with significant differences (raw p-values and corrected p-values (Supplementary Figure 2 D–F). Corresponding data are provided in Supplementary Table 1. Within this report, however, we decided to focus on proteins present exclusively either in MIH patients (Table 1) or healthy controls (Table 2).

### Gene-Ontology Reactome Pathways MIH Saliva

Significantly enriched Gene-Ontology (GO)-terms for control-only proteins (Fig. 1C, upper panel) and patient-only proteins (Fig. 1C, lower panel) are reported. In patient-only proteins, Reactome Pathways (Fig. 1D) showed the highest enrichment score for “neutrophil degranulation” (HSA-6798695; 20 out of 471 (20/471) proteins consolidated for this pathway, followed by “innate immune system (HSA-168249; 25/1012)” and “immune system (HSA-168256; 33/1925)”. Proteins present in these pathways include ACPP, ALAD, CALML5, CD44, CD55, CDA, FTH1, FUCA1, GCA, GMFG, ITGB2, LAMP1, ORM2, PSMA2, PSMB1, PYGB, QPCT, RAB6A, RAP1B, RNASE3. Moreover, proteins accountable for “Platelet activation, signaling and aggregation” (HSA-76002; 6/256), including CD109, CD9, F2, ORM2, RAP1B, TMSB4X and more generally annotated to “Adaptive immune system” (HSA-1280218, 10/733), including BLMH, CANX, ITGB2, PSMA2, PSMB1, RAP1B, SAR1B, TCEB2, UBE2K, VASP were found in MIH-saliva only. In addition, we found proteins annotated to “Cytosolic tRNA aminoacylation” (HSA-379716; 3/24) including GARS, TARS, WARS and proteins (CCT5 and TCP1) implicated in “Folding of actin by CCT/TriC” (HSA-390450; 2/10). Notably, proteins involved in interleukin-1 family signaling (HSA-446652; 4/134), including IL18, IL36RN, PSMA2, and PSMB1, were exclusively found in MIH-saliva.

### Gene-Ontology Biological Process (GOBP), Molecular Function (GOMF), KEGG Pathways and String

Consistently, highest enrichment for Biological Process (GOBP) revealed leukocyte mediated immunity (25/632), immune effector process (26/927), neutrophil mediated immunity (21/498), and neutrophil activation (21/497) – with a comparable signature to the Reactome Pathway “neutrophil degranulation” (HSA-6798695; 20/471). Molecular Function (GOMF) enrichment revealed “catalytic activity” GO:0003824; 43/5592) and “hydrolase activity” GO:0016787; 26/2448), the latter represented by proteins ACPP, BLMH, C11orf54, CAPN2, CDA, CFI, CLC, CTBS, ENOPH1, ESD, F2, FUCA1, GARS, GCA, KLK8, LHPP, NUDT5, OTUB1, PGLYRP2, PREP, PSMA2, PSMB1, RAB6A, RAP1B, RNASE3 and SAR1B in our dataset. Analysis on the basis of the Kyoto Encyclopedia of Genes and Genomes (KEGG) (Fig. 1D) revealed enrichment for “Complement and coagulation cascades” (hsa04610; 4/78) based on the presence of CD55, CFI, F2, ITGB2. Independent of GO analysis, we observed an accumulation of immunoglobulin heavy constant (IGHD) and variable IGHV1–69 IGHV3–15 regions as well as immunoglobulin kappa and lambda variables (IGKV1–5, IGKV1D-39, IGKV2D-24, IGKV4–1, IGLV8–61). Among the chemokines and cytokines, only CCL28, IL18 and the IL36RN was detected in the saliva of MIH patients. STRING interaction networks revealed 78 nodes with an average node degree of 2.51 and molecular links between RAB6A and RAP1B; PSMB1 and PSMA2, RBMX and HNRNPA1, as well as TCP1 and CCT5.

Among the 16 control-only proteins, only CHIT1, CTSA, NAGA were enriched in Molecular Function for “hydrolase activity, hydrolyzing O-glycosyl compounds” (GO:0004553; 3/99). Analysis on the basis of the KEGG revealed HCLS1 and TUBA4A to be enriched for Pathogenic *Escherichia coli* infection (hsa05130; 2/53) and Tight junction (hsa04530; 2/167). CD63, CTSA, and NAGA are enriched in „Lysosome” (hsa04142; 3/123).

## Discussion

Here, we performed a mapping of the proteome of MIH saliva and respective controls from healthy individuals. Our findings show that out of 618 proteins, 88 and 16 proteins were exclusively detected in MIH saliva and control saliva, respectively. Proteins present exclusively in patient´s saliva were functionally linked to “neutrophil degranulation” with the highest enrichment score. In line, enrichment for Biological Process revealed “leukocyte mediated immunity”, “neutrophil mediated immunity” and “neutrophil activation”. Together, these analysis are indicative of ongoing activation and neutrophil degranulation, and supportive of the observed subclinical pulpal inflammation^9^, enhanced emigration of neutrophils into the inflamed pulp^10,11^ and increased numbers of degranulated neutrophils in periodontitis patients^14^. It is thus likely that “neutrophil degranulation” is a confounding element of the salivary protein signature of MIH patients, reflecting ongoing inflammation. Thus, the disease specific signature we identified provides insight into MIH disease pathophysiology and present a potential basis for therapeutic monitoring.

Molecular Function analysis revealed significant enrichment of “catalytic activity” and “hydrolase activity” involving 43 and 26 proteins, covering 50% of the identified proteins in MIH saliva. Catalytic and hydrolase activities are associated with inflammatory processes including neutrophil degranulation, which is linked to tissue degeneration. In this regard, for example, prolyl endopeptidase (PREP), which is produced by neutrophils and cleaves collagen, thereby generating a neutrophil chemoattractant environment, may serve as a valuable biomarker and therapeutic target for diseases caused by chronic, neutrophilic inflammation^53^. Concordantly, interfering with proteolytic activities of the non-lysosomal thiol protease calpain-2 (CAPN2), present exclusively in MIH saliva, could potentially limit the ongoing tissue/bone degradation as calpain-2 inhibitor(s) reportedly reduce colitis and colitis-associated cancer through limiting macrophage activation and inhibiting growth of cancer cells^54^. We identified several proteins in MIH saliva associated with skin-abnormalities caused by chronic inflammation. For example, FUCA1 is a carbohydrate degrading enzyme and FUCA1 gene-mutations are linked to fucosidosis that causes severe skin abnormalities due to disturbed carbohydrate metabolism^55^. The human kallikrein 8 protein (KLK8) is expressed in many normal tissues including the salivary gland^56^. KLK8 serum levels are increased in psoriatic arthritis patients^57^ and in the stratum spinosum during murine skin inflammation^58^. Notably, we also found a protein belonging to the peptidoglycan recognition proteins (PGLYRP2) which recognize bacterial peptidoglycan and functions in antibacterial immunity and inflammation. PGLYRP2 is reportedly produced by salivary glands^59^ and its expression is upregulated by oral epithelial cells derived IL-36 cytokines in response to *Porphyromonas gingivalis* infections^60^. Though we did not detect the PGLYRP2 activating cytokine IL-36 in MIH-saliva, we found an antagonist of this signaling pathway (IL36RN), suggesting counterbalancing feedback mechanisms of this pathway at the receptor-ligand level^60^.

Negative feedback mechanisms limiting inflammation might also operate at the level of the proteasome as we identified proteasome subunits including PSMA2, functionally linked to inflammatory bowel disease^61^ and PSMB1, described to suppresses innate antiviral immunity^62^. Additionally, we identified proteins exerting both pro-and anti-inflammatory properties in different cell types such as the GTPases RAB6A, RAP1B and SAR1B that regulate intracellular protein transport and secretion. While RAB6A facilitates TNF secretion following LPS stimulation of macrophages^63^, RAP1B limits neutrophil tissue infiltration in mice^64^. SAR1B reportedly protects intestinal cells from disorders of lipid homeostasis, oxidative stress, and inflammation^65^. Importantly, we found a remarkable accumulation of immunoglobulins in MIH saliva, a cardinal sign of inflammation. Summarizing, the protein signature of MIH patients is characteristic of other oral inflammatory diseases reflecting an overall principle rather than a disease specific pattern.

Among the chemokines and cytokines, CCL28, IL18 and IL36RN were exclusively identified in the saliva of MIH patients. CCL28 is produced by the salivary gland and displays strong homing capabilities for lymphocytes at mucosal and epithelial sites^66^. CCL28 is not detected in salivary glands of primary Sjögren’s syndrome patients^67^. The stress induced cytokine IL-18 is also produced in salivary glands^68^ and is prominent in the saliva of patients with oral lichen planus^69^ and periodontitis^70^. Proteomic analysis has identified IL-18 as a biomarker in the saliva of burning mouth syndrome, a chronic pain disorder defined by a severe burning sensation in normal looking oral mucosa^71^. IL-36 can act on keratinocytes and immune cells to induce a robust inflammatory response and has been implicated in psoriatic disorders^72^ as well as in inflammatory activation of oral epithelial cells^60^. Supportive of our hypothesis that increased expression of negative regulators mirrors active and ongoing inflammation and in line with our data, IL-36Ra/IL-36RN levels are reported to be higher in active versus inactive ulcerative colitis^73^. Together, proteomic profiles of MIH saliva point towards oral inflammation which is driven by neutrophil activation and degranulation.

Among the 16 control-only proteins enriched in healthy saliva, we identified chitotriosidase (CHIT1) an enzyme with the capacity to hydrolyse chitin, a structural component of fungi, parasitic nematodes, and insects^74^. CHIT1 might have a protective role against chitin-containing pathogens that is absent in MIH saliva. Lysosomal serine carboxypeptidase cathepsin A (CTSA) facilitates the activation of beta-galactosidase and alpha-neuraminidase. Further, CTSA plays a role in the inactivation of bioactive peptides including bradykinin, substances P, oxytocin, angiotensin I and endothelin-I^75^. Alpha-N-acetylgalactosaminidase enzyme (α-NAGA) belongs to the glycoside hydrolase family 27 that breaks down its substrates via the cleavage of their terminal N-acetylgalactosamine residues^76^. Thus, these aforementioned hydrolases might play a role in maintaining innate immune function in normal saliva. Intuitively, their absence in MIH patients makes sense.

In line with this concept, KEGG enrichment revealed HCLS1 and TUBA4A to be enriched for Pathogenic *Escherichia coli* infection (hsa05130; 2/53) and Tight junction (hsa04530; 2/167). For example, hematopoietic cell-specific protein-1 (HCLS1) regulates leukocyte actin remodeling and thereby their recruitment to sites of inflammation^77^. Further indicative of cytoskeletal effects, TUBA4A was enriched, however its role in oral health remains unclear^78^. CD63, a member of the transmembrane 4 superfamily, is a cell-surface protein often used as a marker for multivesicular bodies such as endosomes, lysosomes and exosomes^79,80^, and is interestingly only present in normal saliva but not MIH saliva. Studying exosome in saliva also would apply for MIH patients as proposed for periodontitis^81^, head and neck squamous cell carcinoma^82^ and as liquid biopsy in cancer detection and therapy response prediction^83^.

Our study has limitations. Firstly, we cannot identify the cellular origin of the proteins specifically observed in the MIH saliva nor can we ascribe if the MIH specific protein signature is a cause or consequence of the disease. Hypothesizing, it is plausible that the saliva of MIH patients changes before the disease onset and is caused by an altered physicochemical environment including flow rates and pH-buffering capacities consequent to an accumulation and/or activation of neutrophils that perpetuate MIH- pathogenesis. Another limitation is that we have focused on proteins that are exclusively present in MIH saliva. Differentially expressed proteins would have refined the overall picture of MIH versus healthy patients’ saliva. This kind of analyses is subject of our further investigations and will be reported at a later timepoint. Finally, we are aware of the limitations associated with the sample collection method used. Our study was based on the Salivette system^43^ and there are possible variances when using passive drooling, paraffin gum and Salivette based collection methods^84^. There are also other systems available for saliva collection that may affect result outcome such as Pure·SAL from Oasis Diagnostics^41^ or Sorbette from Becton Dickinson^85^. Indeed, there is the concern of selective absorption of proteins to cotton or cellulose-based collection devices such as eosinophil cationic protein as detection of this particular protein was reported to be higher in passive drool compared to saliva collected using these aforementioned methods^85^. Thus, comparability of our results to other studies might be partially affected not only by the intrinsic heterogeneity of individual clinical subjects but also by the saliva collection method utilized. Future work may employ the analysis of endogenous peptides from saliva isolated from the saliva prior to protein precipitation and extraction, enabling a deeper insight in processes involving biologically active peptides. Given the limitations mentioned above, the use of passive drool collected saliva would be an interesting aspect to consider in the future.

Our work paves the path for future studies which we suggest should focus on possible similarities on the MIH saliva proteins signature identified herein with more established chronic inflammatory diseases such as mucositis, periodontitis, pulpitis and also peri-implantitis. It is possible that in these diseases similar neutrophil related proteins are identified. The present research is also a primer for understanding the role of neutrophils in MIH – in particular, if the neutrophils are only a consequence of the disease and mainly reflect chronic inflammation - or possibly also contribute to its pathogenesis. Although, theoretically, local enhanced neutrophil activation might impair the mineralization of the ameloblasts it is unlikely that neutrophils can affect enamel mineralization. It will be further interesting to exploit novel proteogenomics-based bioinformatic tools and investigate to which extent the diversity of the oral microbiome is changed in MIH saliva versus control subjects.

In conclusion, among 618 salivary proteins identified with high confidence, 88 proteins were identified exclusively in MIH patients. Enrichment analysis revealed neutrophil-mediated adaptive immunity, the activation of the classical pathway of complement activation, extracellular matrix degradation, and other processes linked to inflammation. Whether or not this catabolic environment is a consequence of the inflammation that goes along with MIH warrants further analysis.

## Supplementary information

Supplementary information.
