# Supplementary material for: Saliva proteomic patterns in patients with molar incisor hypomineralization
Source: Sci Rep. 2020 May 5;10:7560. doi: 10.1038/s41598-020-64614-z (PMC7200701; doi:10.1038/s41598-020-64614-z)
Supplement: Supplementary file 1 — Supplementary information. [file 41598_2020_64614_MOESM1_ESM.docx]

**Saliva proteomic patterns in patients with molar incisor hypomineralization**

K. Bekes, G. Mitulović, N. Meissner, U. Resch, R. Gruber

**Supplement Figures and Tables**


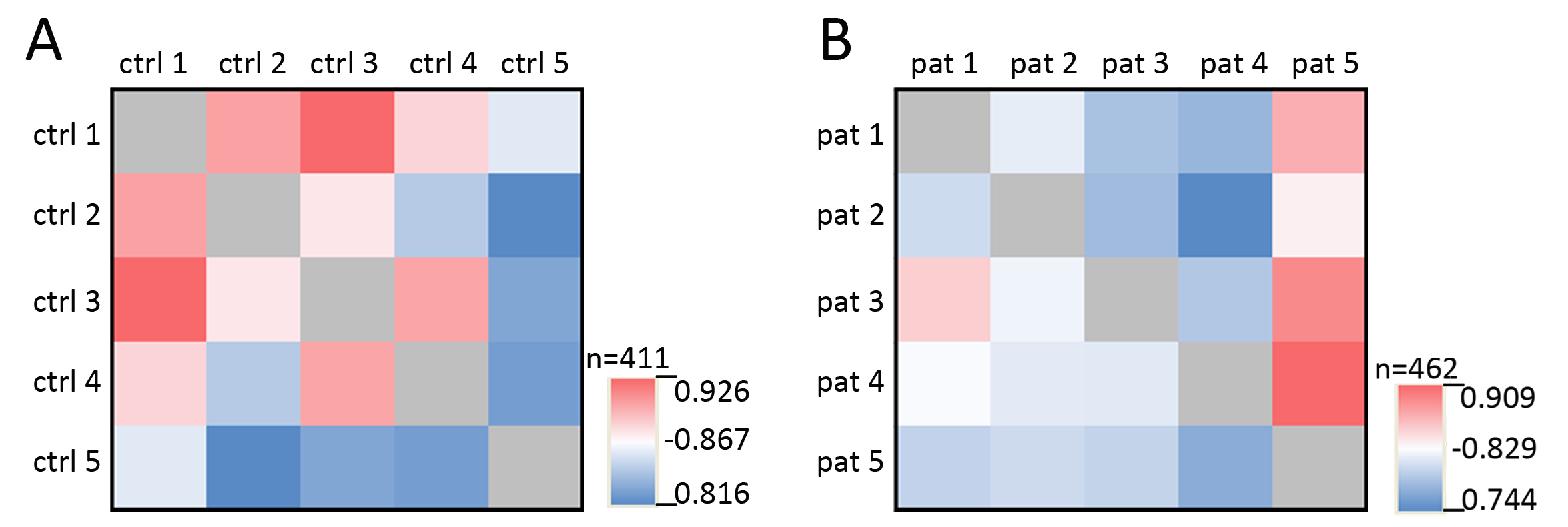


Supplementary Figure 1: Qualitative comparison of saliva proteomes. Interindividual correlations of protein abundance (log2- transformed LFQ-values) in 5 controls (A) and 5 MIH-patients. Color coded Spearman rank-correlation r-values are indicated in the scale bars, total number of proteins used to calculate correlations are indicated at the top of the scale bars.


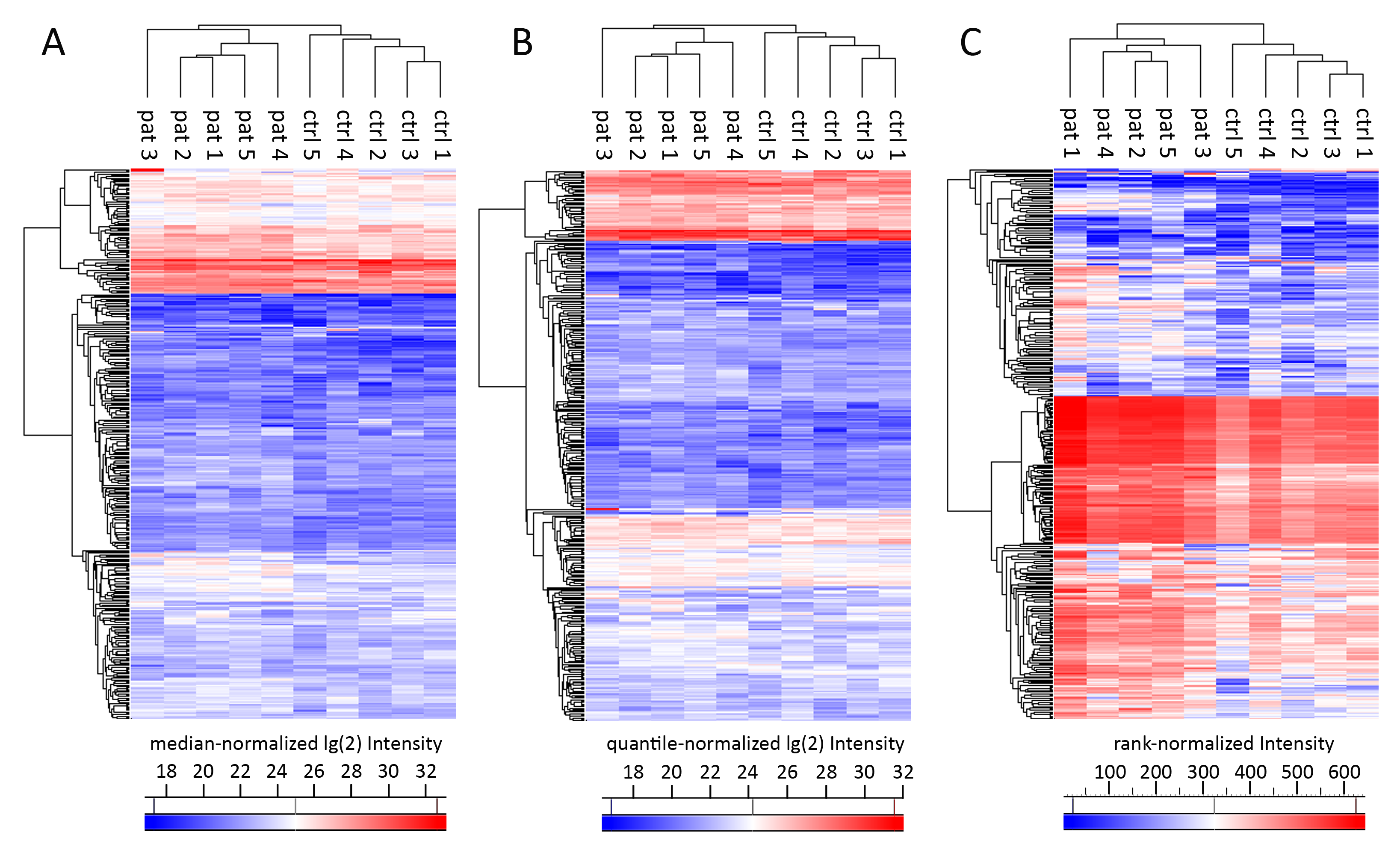

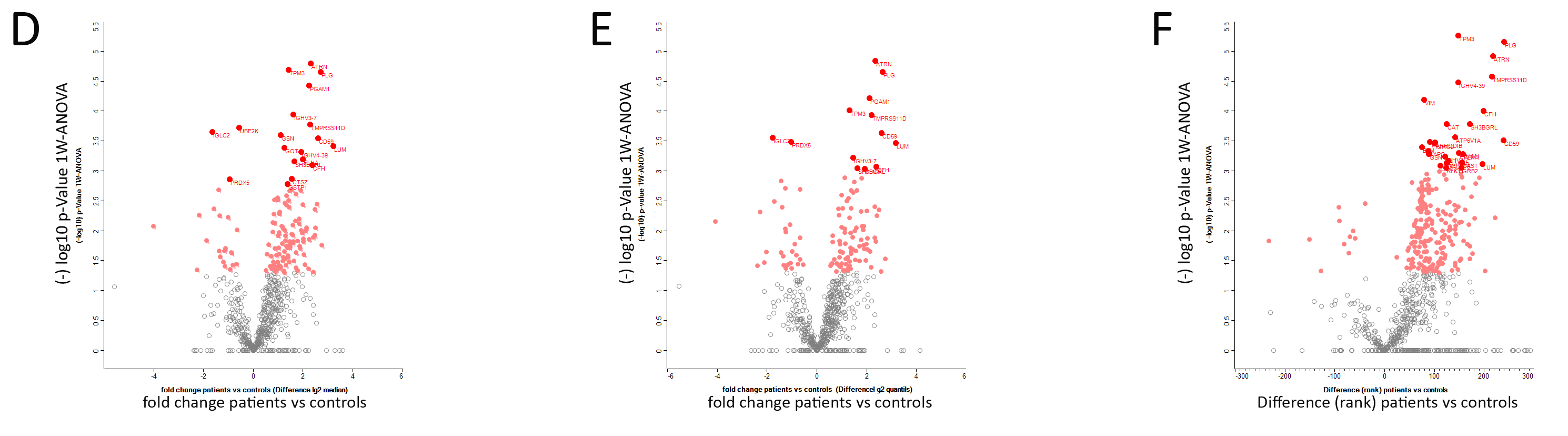


Supplementary Figure 2: Qualitative comparison of saliva proteins in MIH-patients and controls. A-C) Unsupervised hierarchical clustering of LFQ-values after median (A), quantile (B) and rank (C) normalization. Color legends are in indicated on the lower end of respective heatmaps. D-F) Differential expression analysis by one-way-analysis of variance (1W-ANOVA) and graphical representation in Volcano-plots following D) median-, E) quantile- and F) rank-normalization. Significantly different protein abundances (p<0.05) corrected for multiple testing (dark red, gene symbols inscribed) and uncorrected p-values (light red) are indicated.

Supplementary Table 1: Study-cohort characteristics

| **sample** | **sex** | **age (y)** | **sample** | **sex** | **age (y)** | **# of MIH teeth** | **EAPD criteria*** | **TNI** | **Schiff** | **VAS** |
| --- | --- | --- | --- | --- | --- | --- | --- | --- | --- | --- |
|  |  |  |  |  |  |  |  |  |  |  |
| control 1 | m | 10.43 | patient 1 | f | 9.16 | 4 | O/O/pB/pB | 3.50±0.50 | 2.50±0.50 | 5.50±2.60 |
| control 2 | m | 11.02 | patient 2 | m | 6.21 | 4 | O/O/pB/O | 3.25±0.43 | 2.00±0.00 | 7.00±1.00 |
| control 3 | m | 11.37 | patient 3 | f | 10.74 | 4 | pB/pB/O/O | 3.50±0.50 | 2.50±0.50 | 8.00±2.12 |
| control 4 | f | 9.98 | patient 4 | f | 6.27 | 4 | O/O/O/pB | 2.25±1.30 | 1.25±1.30 | 3.50±3.57 |
| control 5 | f | 10.84 | patient 5 | f | 11.12 | 3 | O/pB/pB | 3.00±1.41 | 2.00±1.41 | 5.00±3.56 |
|  |  |  |  |  |  |  |  |  |  |  |
| **mean±sd** |  | **10.73±0.54** | **mean±sd** |  | **8.70±2.36** | **3.80±0.45** |  | **3.10±0.52** | **2.05±0.51** | **5.80±1.75** |

* O = opacity; pB = posteruptive breakdown

**Supplementary Methods**

**Saliva proteomic patterns in patients with molar incisor hypomineralization**

K. Bekes, G. Mitulović, N. Meissner, U. Resch, R. Gruber

**Introduction**

The term molar incisor hypomineralization (MIH) was coined in 2001 to define a qualitative enamel defect that affects one or more first permanent molars with or without the involvement of permanent incisors ([Weerheijm et al. 2001](#_ENREF_33)). The global prevalence exceeds one-tenth of the children, ranging from 0.5% to 40.2% and differing between countries ([Zhao et al. 2018](#_ENREF_35)). Affected enamel displays increased porosity and reduced hardness compared to normal teeth ([Elhennawy et al. 2017](#_ENREF_7)). The clinical management of MIH is challenging because of progressing enamel erosion, increased susceptibility to caries, and severe hypersensitivity ([Lygidakis 2010](#_ENREF_12)). Although MIH is a pandemic pediatric disease, the etiology remains unknown ([Mast et al. 2013](#_ENREF_14)) even though a genetic component is suspected ([Silva et al. 2016](#_ENREF_28); [Taylor 2017](#_ENREF_30)). There is thus an increasing demand to better understand the cause and the consequence of MIH at the cellular and molecular level.

Chronic subclinical pulpal inflammation is a consequence of increased enamel porosity and reduced hardness in MIH ([Dixit and Joshi 2018](#_ENREF_6)). Pulpitis is characterized by enhance emigration of neutrophils in the pulp tissue ([Mente et al. 2016](#_ENREF_16); [Rethnam et al. 2010](#_ENREF_26)) and biomarkers can be identified in gingival crevicular fluid ([Rechenberg et al. 2016](#_ENREF_24)). Moreover, neutrophils constantly migrate through the oral epithelia into the saliva ([Calonius 1958](#_ENREF_3)) with increasing numbers, being more apoptotic and having increased levels of degranulation markers in periodontitis patients ([Nicu et al. 2018](#_ENREF_18)). Also, saliva of MIH patients reportedly displays altered physicochemical properties in terms of flow rates, viscosity, pH and acid buffering capacity ([Ghanim et al. 2013](#_ENREF_9)). In conjunction, it is conceivable that also the protein-composition of MIH saliva exhibits characteristic changes which may cause or contribute to the clinical symptoms of MIH.

Salivary proteome analysis has progressively evolved in the last decade ([Siqueira and Dawes 2011](#_ENREF_29); [Vitorino et al. 2004](#_ENREF_31)). The salivary proteome has been exploited to detect oral diseases such as periodontitis ([Orti et al. 2018](#_ENREF_21)), oral squamous cell carcinoma ([Radhika et al. 2016](#_ENREF_22)),burning mouth syndrome ([Cabras et al. 2019](#_ENREF_1)) as well as Sjögren’s syndrome ([Katsiougiannis and Wong 2016](#_ENREF_10)). Moreover, the saliva proteome was analyzed in systemic diseases, e.g. diabetes mellitus ([Rao et al. 2009](#_ENREF_23)), cystic fibrosis ([Minarowski et al. 2008](#_ENREF_17)), Parkinson disease ([Ren et al. 2015](#_ENREF_25)), pulmonary tuberculosis ([Mateos et al. 2019](#_ENREF_15)), multiple sclerosis ([Manconi et al. 2018](#_ENREF_13)) as well as in psychiatric ([Wormwood et al. 2015](#_ENREF_34)) and genetic diseases ([Cabras et al. 2013](#_ENREF_2)), proving the great potential of proteomics not merely in the identification of biomarkers but also providing insight into pathology underlying mechanisms at the molecular level. As changes in saliva have been described in the presence of oral diseases, it is reasonable to assume that the saliva of children with MIH that experience subclinical inflammation might be altered compared to healthy children. The aim of the present study is to employ high-resolution shotgun proteomics to identify the protein signature that is unique to MIH patients.

**Material and Methods**

*Proteomic analysis*

Saliva proteins were precipitated using methanol/dichloromethane and digested with trypsin as described earlier ([Fichtenbaum et al. 2016](#_ENREF_8)) (For detail see Suppl. Methods 1). Precipitated proteins were dissolved in 0.1% Rapigest (Waters, Vienna, Austria), dissolved in 50mM triethylammonium bicarbonate, and protein concentration was determined using the Bradford assay. Proteins were digested overnight at 37°C using a trypsin:protein ratio of 1:50, digestion was stopped by acidification with trifluoroacetic acid (TFA). Following injection onto the trapping column (Acclaim C18 trap column, 300μm inner diameter × 5 mm), peptides were separated by nano-reverse-phase (Acclaim C18, 75μm inner diameter × 500 mm) using an UltiMate nano RSLC HPLC (Thermo Fisher, Germering, Germany) separation system, consisting of the autosampler, column switching unit, nano and loading pump and UV detector. Both, trap- and separation columns were operated at 60°C and UV peptide detection at 214 nm served as quality control for HPLC separation. Samples were loaded onto the trap column using 0.1% TFA at 30 μl/min and precooled to 3°C ([Schöbinger et al. 2016](#_ENREF_27)), nano separation was performed in gradient mode at 300 nl/min. A user defined injection program was used for sample injection and additional injector and trap column wash. Every sample injection was followed by two blank runs with injections of 2,2,2-trifluoroethanol for removal of possible sample remains in the injector or on the trap column and prevention of carryover in the separation system. Mass spectrometry (MS) analysis was performed using the Q-Exactive plus mass spectrometer (Thermo Fisher Scientific) and the “top 20” method for MS/MS experiment; that is, the 20 most intensive ions from the MS scan were selected for tandem MS (MS/MS), single-charged ions were excluded from fragmentation, and detected ions were excluded for further fragmentation for 2 min after initial MS/MS fragmentation had been performed. Mass resolution of 70000 was selected for MS at AGC set to 3E6, MS/MS resolution was set to 35000 and AGC set to 1E5 scans. Fragmentation was performed using the HCD approach at normalized collision energy of 30eV. Data analysis (database search and label-free quantitation) was performed using MaxQuant (version 1.6.0.1) with following parameters: MS/MS data were searched against the Human Fasta Database (Uniprot, version September 2018), MS/MS tolerance was set to 20ppm, deamidation on N and Q, N-term acetylation and oxidation on M were selected as variable modifications. The mass spectrometry proteomics data have been deposited to the ProteomeXchange Consortium via the PRIDE partner repository with the dataset identifier PXD016126 ([Vizcaino et al. 2016](#_ENREF_32)).

**Statistics**

Bioinformatic analyses of protein identifications were done in Perseus (version 1.5.5.3) and overlapping proteins were visualized in Venn-Diagrams in combination with the CNB-CSIC online tool *Venny* ([Oliveros 2007-2015](#_ENREF_20))*.* Gene-Ontology as well as biological pathway enrichment analysis were done using the “Enricher” interactive data-analysis tool ([Chen et al. 2013](#_ENREF_5); [Kuleshov et al. 2016](#_ENREF_11)). Throughout this report we express enriched terms by enumerating the actual number of proteins in our dataset in relation to the total number of proteins consolidated for the respective pathway. Normalizations were done using the web-based tool NormalyzerDE ([Chawade et al. 2014](#_ENREF_4)). Spearman-rank correlation analysis and visualization of log2-transformed label-free quantification (LFQ) values, unsupervised hierarchical clustering and heat-maps of median, quantile and rank-normalized protein abundance values were done in Perseus using default settings (euclidian-distance on average linkage, pre-processing with k-means with a maximum of 300 clusters and 10 iterations). For group-wise comparisons we employed FDR-based 1-Way-ANOVA with 250 permutations and multiple-testing correction of p-values were done in InstantClue (version 0.5.2) using non-log transformed p-values for 2-stage-set-up Benjamini-Krieger-Yekutieli, alpha-error of 0.05 as well as Benjamini-Hochberg correction methods ([Nolte et al. 2018](#_ENREF_19)). Volcano-plots were prepared in Perseus and proteins with a corrected p-value <0.05 were annotated with the corresponding gene symbol, while proteins with a non-adjusted p-value <0.05 were color-indicated only. For rank-normalization, only proteins with a corrected p-value smaller than 0.015 were annotated. For proteins exclusively present in patients or controls, a (-)log10-p-value of zero was assigned after all statistical analysis to enable visualization in Volcano plots. Visualization of protein–protein associations was performed with STRING v11.

**References**

Cabras T, Manconi B, Castagnola M, Sanna MT, Arba M, Acharya S, Ekstrom J, Carlen A, Messana I. 2019. Proteomics of the acid-soluble fraction of whole and major gland saliva in burning mouth syndrome patients. Arch Oral Biol. 98:148-155.

Cabras T, Pisano E, Montaldo C, Giuca MR, Iavarone F, Zampino G, Castagnola M, Messana I. 2013. Significant modifications of the salivary proteome potentially associated with complications of down syndrome revealed by top-down proteomics. Mol Cell Proteomics. 12(7):1844-1852.

Calonius PE. 1958. The leukocyte count in saliva. Oral Surg Oral Med Oral Pathol. 11(1):43-46.

Chawade A, Alexandersson E, Levander F. 2014. Normalyzer: A tool for rapid evaluation of normalization methods for omics data sets. J Proteome Res. 13(6):3114-3120.

Chen EY, Tan CM, Kou Y, Duan Q, Wang Z, Meirelles GV, Clark NR, Ma'ayan A. 2013. Enrichr: Interactive and collaborative html5 gene list enrichment analysis tool. BMC Bioinformatics. 14:128.

Dixit UB, Joshi AV. 2018. Efficacy of intraosseous local anesthesia for restorative procedures in molar incisor hypomineralization-affected teeth in children. Contemp Clin Dent. 9(Suppl 2):S272-S277.

Elhennawy K, Manton DJ, Crombie F, Zaslansky P, Radlanski RJ, Jost-Brinkmann PG, Schwendicke F. 2017. Structural, mechanical and chemical evaluation of molar-incisor hypomineralization-affected enamel: A systematic review. Arch Oral Biol. 83:272-281.

Fichtenbaum A, Schmid R, Mitulović G. 2016. Direct injection of hilic fractions on the reversed-phase trap column improves protein identification rates for salivary proteins. ELECTROPHORESIS. 37(22):2922-2929.

Ghanim A, Marino R, Morgan M, Bailey D, Manton D. 2013. An in vivo investigation of salivary properties, enamel hypomineralisation, and carious lesion severity in a group of iraqi schoolchildren. Int J Paediatr Dent. 23(1):2-12.

Katsiougiannis S, Wong DT. 2016. The proteomics of saliva in sjogren's syndrome. Rheum Dis Clin North Am. 42(3):449-456.

Kuleshov MV, Jones MR, Rouillard AD, Fernandez NF, Duan Q, Wang Z, Koplev S, Jenkins SL, Jagodnik KM, Lachmann A et al. 2016. Enrichr: A comprehensive gene set enrichment analysis web server 2016 update. Nucleic Acids Res. 44(W1):W90-97.

Lygidakis NA. 2010. Treatment modalities in children with teeth affected by molar-incisor enamel hypomineralisation (mih): A systematic review. Eur Arch Paediatr Dent. 11(2):65-74.

Manconi B, Liori B, Cabras T, Vincenzoni F, Iavarone F, Lorefice L, Cocco E, Castagnola M, Messana I, Olianas A. 2018. Top-down proteomic profiling of human saliva in multiple sclerosis patients. J Proteomics. 187:212-222.

Mast P, Rodrigueztapia MT, Daeniker L, Krejci I. 2013. Understanding mih: Definition, epidemiology, differential diagnosis and new treatment guidelines. Eur J Paediatr Dent. 14(3):204-208.

Mateos J, Estevez O, Gonzalez-Fernandez A, Anibarro L, Pallares A, Reljic R, Gallardo JM, Medina I, Carrera M. 2019. High-resolution quantitative proteomics applied to the study of the specific protein signature in the sputum and saliva of active tuberculosis patients and their infected and uninfected contacts. J Proteomics. 195:41-52.

Mente J, Petrovic J, Gehrig H, Rampf S, Michel A, Schurz A, Pfefferle T, Saure D, Erber R. 2016. A prospective clinical pilot study on the level of matrix metalloproteinase-9 in dental pulpal blood as a marker for the state of inflammation in the pulp tissue. J Endod. 42(2):190-197.

Minarowski L, Sands D, Minarowska A, Karwowska A, Sulewska A, Gacko M, Chyczewska E. 2008. Thiocyanate concentration in saliva of cystic fibrosis patients. Folia Histochem Cytobiol. 46(2):245-246.

Nicu EA, Rijkschroeff P, Wartewig E, Nazmi K, Loos BG. 2018. Characterization of oral polymorphonuclear neutrophils in periodontitis patients: A case-control study. BMC Oral Health. 18(1):149.

Nolte H, MacVicar TD, Tellkamp F, Kruger M. 2018. Instant clue: A software suite for interactive data visualization and analysis. Sci Rep. 8(1):12648.

Oliveros JC. 2007-2015. Venny-an interactive tool for comparing lists with venn's diagrams.

Orti V, Mertens B, Vialaret J, Gibert P, Relano-Gines A, Lehmann S, Deville de Periere D, Hirtz C. 2018. Data from a targeted proteomics approach to discover biomarkers in saliva for the clinical diagnosis of periodontitis. Data Brief. 18:294-299.

Radhika T, Jeddy N, Nithya S, Muthumeenakshi RM. 2016. Salivary biomarkers in oral squamous cell carcinoma - an insight. J Oral Biol Craniofac Res. 6(Suppl 1):S51-S54.

Rao PV, Reddy AP, Lu X, Dasari S, Krishnaprasad A, Biggs E, Roberts CT, Nagalla SR. 2009. Proteomic identification of salivary biomarkers of type-2 diabetes. J Proteome Res. 8(1):239-245.

Rechenberg DK, Galicia JC, Peters OA. 2016. Biological markers for pulpal inflammation: A systematic review. PLoS One. 11(11):e0167289.

Ren R, Sun Y, Zhao X, Pu X. 2015. Recent advances in biomarkers for parkinson's disease focusing on biochemicals, omics and neuroimaging. Clin Chem Lab Med. 53(10):1495-1506.

Rethnam S, Raju B, Fristad I, Berggreen E, Heyeraas KJ. 2010. Differential expression of neuropeptide y y1 receptors during pulpal inflammation. Int Endod J. 43(6):492-498.

Schöbinger M, Klein OJ, Mitulović G. 2016. Low-temperature mobile phase for peptide trapping at elevated separation temperature prior to nano rp-hplc–ms/ms. Separations. 3:6.

Silva MJ, Scurrah KJ, Craig JM, Manton DJ, Kilpatrick N. 2016. Etiology of molar incisor hypomineralization - a systematic review. Community Dent Oral Epidemiol. 44(4):342-353.

Siqueira WL, Dawes C. 2011. The salivary proteome: Challenges and perspectives. Proteomics Clin Appl. 5(11-12):575-579.

Taylor GD. 2017. Molar incisor hypomineralisation. Evid Based Dent. 18(1):15-16.

Vitorino R, Lobo MJ, Ferrer-Correira AJ, Dubin JR, Tomer KB, Domingues PM, Amado FM. 2004. Identification of human whole saliva protein components using proteomics. Proteomics. 4(4):1109-1115.

Vizcaino JA, Csordas A, Del-Toro N, Dianes JA, Griss J, Lavidas I, Mayer G, Perez-Riverol Y, Reisinger F, Ternent T et al. 2016. 2016 update of the pride database and its related tools. Nucleic Acids Res. 44:D447.

Weerheijm KL, Jalevik B, Alaluusua S. 2001. Molar-incisor hypomineralisation. Caries Res. 35(5):390-391.

Wormwood KL, Aslebagh R, Channaveerappa D, Dupree EJ, Borland MM, Ryan JP, Darie CC, Woods AG. 2015. Salivary proteomics and biomarkers in neurology and psychiatry. Proteomics Clin Appl. 9(9-10):899-906.

Zhao D, Dong B, Yu D, Ren Q, Sun Y. 2018. The prevalence of molar incisor hypomineralization: Evidence from 70 studies. Int J Paediatr Dent. 28(2):170-179.
